# Application of biomarkers in the diagnosis of uncertain samples of core needle biopsy of thyroid nodules

**DOI:** 10.1007/s00428-021-03161-y

**Published:** 2021-07-26

**Authors:** Yan Xiong, Xin Li, Li Liang, Dong Li, Limin Yan, Xueying Li, Jiting Di, Ting Li

**Affiliations:** 1 Department of Pathology, Peking University First Hospital, 7 Xishiku Street, Xicheng District, Beijing, 100034 China; 2 Department of Pathology, Tangshan Gongren Hospital, 27 Wenhua Road, Lubei District, Tangshan, 063000 Hebei China; 3 Department of Biostatistics, Peking University First Hospital, 7 Xishiku Street, Xicheng District, Beijing, 100034 China

**Keywords:** Thyroid nodule, Core needle biopsy, Uncertain, Biomarker

## Abstract

Core needle biopsy (CNB) is now more frequently used for the preoperative diagnosis of thyroid nodules. Based on morphology alone, 5–20% of CNB samples cannot be determined as malignant or benign. Compared to fine-needle biopsy (FNB), samples collected by CNB are more accessible for various tests. Therefore, studying biomarkers’ application in distinguishing uncertain CNB samples of thyroid nodules is a practical need. Patients of thyroid nodules with both CNB and matched resected specimens were reviewed. Cases classified as indeterminate lesions, follicular neoplasms, and suspicious for malignancy were retrieved. All CNB samples were stained by immunohistochemistry (IHC) using antibodies against CK19, galectin-3, HBME-1, and CD56 and detected by next-generation sequencing (NGS) using an OncoAim® thyroid cancer multigene assay kit (Singlera Genomics) that detected 26 genes. Taking the resected specimens’ classification as the gold standard, the sensitivity, specificity, positive predictive value (PPV), negative predictive value (NPV), accuracy of a single biomarker, and various combinations for discriminating malignancy from benignity were calculated. The sensitivity, specificity, PPV, NPV, and accuracy for preoperative malignancy evaluation were as follows. In the cohort of non-follicular-neoplasm-lesions (non-FN-lesion), they were 95.16%, 53.85%, 90.77%, 70.00%, and 88.00% for CK19; 95.16%, 38.46%, 88.06%, 62.50%, and 85.33% for galectin-3; 77.42%, 76.92%, 94.12%, 41.67%, and 58.00% for HBME-1; 66.13%, 100.00%, 100.00%, 38.24%, and 72.00% for CD56; 90.32%, 92.31%, 98.25%, 66.67%, and 90.67% for NGS; and 88.71%, 92.30%, 98.21%, 63.16%, and 89.33% for integrated IHC. In the cohort of follicular neoplasms (FN), they were 30.43%, 77.77%, 77.77%, 30.43%, and 43.75% for CK19; 73.91%, 66.67%, 85.00%, 50.00%, and 71.88% for galectin-3; 26.09%, 88.89%, 85.71%, 32.00%, and 43.75% for HBME-1; 26.09%, 100.00%, 100.00%, 34.62%, and 46.88% for CD56; 52.17%, 88.89%, 92.31%, 42.11%, and 62.50% for NGS; 82.61%, 66.67%, 86.36%, 60.00%, and 78.13% for integrated IHC; and 100%, 66.67%, 88.46%, 100%, and 90.63% for integrated IHC-NGS. The application of biomarkers in distinguishing uncertain CNB samples of thyroid nodules is available and capable. CD56 negative or NGS positive suggests malignancy strongly for both FN and non-FN-lesion, which may be used as a “rule in” tool. The negative predictive value of the integrated IHC and the integrated IHC-NGS implies a high possibility to be benign for non-FN-lesion and FN separately, which can work as a “rule out” tool. Considering the balance of specificity and sensitivity, NGS is the best for non-FN-lesion and the integrated IHC-NGS is the best for FN.

## Background

Thyroid nodules are a common disease of the endocrine system. The prevalence is 20 to 76% in the Chinese population as identified by high-resolution ultrasound, and 5 to 15% of nodules are malignant [1]. It is crucial to screen these malignant cases for further treatment. The biopsy techniques involved in thyroid nodules’ preoperative diagnosis include fine-needle biopsy (FNB) and core needle biopsy (CNB). FNB has been used worldwide for many years, and CNB has been used more and more frequently in Asia in the recent 10 years [2, 3]. Several large single-center studies have shown no significant differences between FNB and CNB in terms of pain, tolerability, or complications due to the advances in CNB devices and the development of high-resolution ultrasound [4]. Compared to FNB, the morphology of cells and architectures of the tumors can be seen in the CNB samples, giving more support to pathologists to make a correct diagnosis. Published studies have shown that the accuracy of CNB for the thyroid nodules’ preoperative diagnosis was higher than of FNB [5]. However, approximately 5–20% of CNB samples are still uncertain of being benign or malignant based on morphology alone [5, 6]. Compared to FNB, samples collected by CNB are more accessible for various testing methods. Therefore, studying biomarkers’ application in distinguishing uncertain CNB samples of thyroid nodules is a practical need. We retrieved 107 cases of thyroid nodules with uncertain CNB samples and matched resected specimens. Taking the matched resected specimens’ diagnosis as the gold standard, we studied biomarkers’ capability to distinguish uncertain CNB samples.

## Methods

### Patients and samples

Patients of thyroid nodules with both CNB and matched resected specimens treated at Peking University First Hospital between January 2015 and December 2020 were reviewed. CNB was used as the first-line preoperative diagnosis in all patients without prior FNB according to publication protocol [7]. The Peking University First Hospital Ethics Committee approved the usage of all patient samples and clinical data and an informed consent exemption (ethical approval no.: (2018) Research No. 147).

### Pathological review

All hematoxylin and eosin (HE) staining slides were separately reviewed by two pathologists blinded to the original diagnoses. The CNB samples were diagnosed according to the Korean proposal: (I) nondiagnostic or unsatisfactory; (II) benign lesion; (III) indeterminate lesion; (IV) follicular neoplasm; (V) suspicious for malignancy; and (VI) malignant (Table 1) [8]. Cases classified as III–V were retrieved as “uncertain.” The resected samples were diagnosed according to the 2017 WHO classification of tumors of endocrine organs (4th): conventional papillary thyroid carcinoma (CPTC), follicular variant papillary thyroid carcinoma (FVPTC), follicular thyroid carcinoma (FTC), follicular adenoma (FA), nodular hyperplasia (NH), and thyroiditis [9]. The cases with inconsistent diagnoses were reviewed, and agreements were achieved by discussion. Furthermore, we divided the cohort into two groups, i.e., the follicular neoplasm (FN) and the non-follicular-neoplasm-lesion (non-FN-lesion), to see if the biomarkers’ efficiency was different. The FN included FTC and FA. The non-FN-lesion included CPTC, FVPTC, NH, and thyroiditis.

**Table 1:** Diagnostic categories of thyroid core needle biopsy proposed by the Korean Thyroid Association [5]

I. Nondiagnostic or unsatisfactory
• Non-tumor adjacent thyroid tissue only
• Extrathyroid tissue only (e.g., skeletal muscle, mature adipose tissue)
• Acellular specimen (e.g., acellular fibrotic tissue, acellular hyalinized tissue, cystic fluid only)
• Blood clot only
• Other
II. Benign lesion
• Benign follicular nodule
• Hashimoto’s thyroiditis
• Subacute granulomatous thyroiditis
• Nonthyroidal lesion (e.g., parathyroid lesions, benign neurogenic tumors, benign lymph node)
• Other
III. Indeterminate lesion
IIIa. Indeterminate follicular lesion with nuclear atypia
IIIb. Indeterminate follicular lesion with architectural atypia
IIIc. Indeterminate follicular lesion with nuclear and architectural atypia
IIId. Indeterminate follicular lesion with Hürthle cell changes
IIIe. Indeterminate lesion, not otherwise specified
IV. Follicular neoplasm
IVa. Follicular neoplasm, conventional type
IVb. Follicular neoplasm with nuclear atypia
IVc. Hürthle cell neoplasm
IVd. Follicular neoplasm, not otherwise specified
V. Suspicious for malignancy
• Suspicious for papillary carcinoma, medullary carcinoma, poorly differentiated carcinoma, metastatic carcinoma, lymphoma, etc
VI. Malignant
• Papillary thyroid carcinoma, poorly differentiated carcinoma, anaplastic thyroid carcinoma, medullary thyroid carcinoma, lymphoma, metastatic carcinoma, etc

### Immunohistochemistry stain

The primary antibodies included antibodies against CK19 (Dako, Clone RCK108), galectin-3 (Invitrogen, A3A12), HBME-1 (Dako, Clone HBME-1), and CD56 (Dako, Clone 123C3). The antigen retrieval buffer was EDTA (pH 9.0), the temperature was 98 °C, and the duration was 20 min. We used EnVision FLEX + Mouse LINKER to amplify the signal, the EnVision FLEX Mini Kit to visualize the immunohistochemistry (IHC) reaction, and the Autostainer Link 48 (Agilent Technologies, Santa Clara, CA, USA) to complete the procedure. The normal thyroid follicles around the nodules were the best IHC staining and evaluation controls for CD56. For CK19, galectin-3, and HBME-1, the known positive samples were put side by side with the target samples on each slide as controls.

### Scoring the results of a single IHC biomarker

Tumors with membranous ± cytoplasmic reactivity for CK19 in more than 10% of cells with strong intensity were considered positive. Tumors with cytoplasmic + nuclear reactivity for galectin-3 and membranous reactivity for HBME-1 or CD56 in more than 10% of cells were deemed positive regardless of intensity [10].

### Integrating IHC markers

The cohort positive of integrated IHC consisted of two groups: The first was CD56 negative no matter whether CK19, galectin-3, and HBME-1 were stained or not; The second was CD56 positive and the other markers simultaneously positive. The cutoff of simultaneously positive markers was different in the different panels. The first panel, named IHC-COMB1, required all three simultaneously positive; the second panel, named IHC-COMB2, required at least two, and the third panel, named IHC-COMB3, required at least one.

### Next-generation sequencing

The percentage of tumor components in the CNB samples was recorded. Genomic DNA was extracted from unstained 5-µm-thick paraffin-embedded sections using the QIAamp DNA FFPE Tissue Kit (Qiagen, Hilden, Germany) following the manufacturer’s instructions. After extraction, DNA quality was evaluated by 1% agarose gel electrophoresis. The concentration of all samples was quantitated by a NanoDrop system (Invitrogen Life Technologies, Carlsbad, CA, USA) and Qubit Fluorometer (Invitrogen Life Technologies).

Targeted next-generation sequencing (NGS) was conducted using an OncoAim® thyroid cancer multigene assay kit (Singlera Genomics, Inc., Shanghai, China) that detected 26 genes (Table 2). According to the kit protocol, 50 ng of DNA for each sample was used to generate sequencing libraries. DNA was fragmented by 5 × WGS Fragmentation Mix (Qiagen, Beverly, MA, USA). After quality control and quantification, the library product was sequenced using 150 bp paired-end runs on the NextSeq 500 platform (Illumina, Inc., San Diego, CA, USA). Sequencing data were then aligned to the reference human genome (hg19). Read mapping, quality control, variant calling, and genotyping were performed automatically using the tool kit supplied in the OncoAim® Kit (Singlera). The minimum confidence threshold for variant calling was set to 5%. Variant functional annotation was performed with the ENSEMBL Variant Effect Predictor tool.

**Table 2:** Genes detected by OncoAim® thyroid cancer multigene assay kit

Gene	Transcript	Variation type	
		Mutation	Fusion
*BRAF*	NM_004333	Exon 15	Introns 7–10
*RET*	NM_020975	Exons 7–16	Introns 10–11
*NRAS*	NM_002524	Exons 2–3	-
*KRAS*	NM_033360	Exons 2–4	-
*HRAS*	NM_176795	Exons 2–3	-
*AKT1*	NM_005163	Exons 2–7, exons 9–12	-
*ATM*	NM_000051	All exons	-
*CNNB1*	NM_001904	All exons	-
*TSHR*	NM_000369	All exons	-
*APC*	NM_000038	All exons	-
*TTN*	NM_001256850	All exons	-
*TG*	NM_003235	All exons	-
*RB1*	NM_000321	All exons	-
*MEN1*	NM_000244	All exons	-
*PDGFRA*	NM_006206	All exons	-
*PIK3CA*	NM_006218	All exons	-
*CDKN2A*	NM_000077	All exons	-
*EIF1AX*	NM_001412	All exons	-
*PTEN*	NM_000314	Exons 5–8	-
*GNAS*	NM_000516	Exons 8–9	-
*TP53*	NM_000546	Exons 5–9	-
*TERT*	*NM_198253*	Promoter (chr5:1,295,183–1,295,302)	-
*PPARG*	NM_005037	-	Intron 1
*NTRK1*	NM_002529	-	Intron 9, exon 12
*NTRK3*	NM_002530	-	Intron 13
*ALK*	NM_004304	-	Intron 16, intron 19

Based on ClinVar (Version 20,280,919), the result was marked as pathogenic, likely pathogenic, uncertain significance, likely benign, benign, or inconclusive. We recorded “confirmed pathogenic” or “likely pathogenic” as NGS positive.

### Integrating IHC and NGS

The cohort positive of integrated IHC-NGS consisted of two groups: The first was NGS positive no matter whether the IHC markers were stained or not; The second was NGS negative and at least one of four IHC markers positive.

### Comparison between biomarkers’ results of CNB samples and classification of matched resected specimens

The results of biomarkers detected on CNB samples were compared to the classification of matched resected specimens.

### Statistical analysis

We put resected samples classified as CPTC, FVPTC, and FTC into a single group as “malignant” and thyroiditis, NH, and FA into another group as “benign” for statistical analysis. Taking the resected specimens’ classification as the gold standard, the sensitivity, specificity, positive predictive value (PPV), negative predictive value (NPV), and accuracy of each biomarker and various integrated panels for discriminating malignancy from benignity were calculated.

## Results

### Patients

The study included 107 patients. Of them, 27 were males and 80 were females, with ages ranging from 20 to 82 and a mean age of 50. Sixty-five patients were younger than 55, thirty-nine were older than 55, and three were 55.

### Histological classification

The CNB samples included 40 (37.4%) cases of indeterminate, 32 (29.9%) cases of follicular neoplasm, and 35 (32.7%) cases of suspicious malignancy.

Twenty-two (20.6%) resected specimens were classified as benign, including 9 (8.4%) cases of NH, 4 (3.7%) cases of thyroiditis, and 9 (8.4%) cases of FA. Eighty-five (79.4%) resected specimens were classified as malignant, including 35 (32.7%) cases of CPTC, 27 (25.2%) cases of FVPTC, and 23 (21.5%) cases of FTC.

Of the 40 cases classified as indeterminate on CNB samples, the matched resected samples were classified as thyroiditis for 4 cases, NH for 9 cases, and FVPTC for 27 cases. Of the 32 cases classified as follicular neoplasm on CNB samples, the matched resected samples were classified as FA for 9 cases and FTC for 23 cases. All 35 cases classified as suspicious malignancy on CNB samples were classified as CPTC on resected samples (Table 3).

**Table 3:** Comparison between classification of CNB samples and classification of matched resected specimens based on morphology alone

Classification of resected samples	Classification of CNB samples based on morphology, no			
	Indeterminate lesion	Follicular neoplasm	Suspicious for malignancy	Total
NH	9	0	0	9
Thyroiditis	4	0	0	4
FA	0	9	0	9
CPTC	0	0	35	35
FVPTC	27	0	0	27
FTC	0	23	0	23
Total	40	32	35	107

*CNB*, core needle biopsy; *NH*, nodular hyperplasia; *FA*, follicular adenoma; *CPTC*, conventional papillary thyroid carcinoma; *FVPTC*, follicular variant of papillary thyroid carcinoma; *FTC* follicular thyroid carcinoma

### Results of IHC

Seventy-four cases (69.16%) were positive for CK19, including 31 cases of indeterminate lesion, 9 cases of follicular neoplasm, and 34 cases of suspicious malignancy. Eighty-seven cases (81.31%) were positive for galectin-3, including 33 cases of indeterminate lesion, 20 cases of follicular neoplasm, and 34 cases of suspicious malignancy. Fifty-eight cases (54.21%) were positive for HBME-1, including 24 cases of indeterminate lesion, 7 cases of follicular neoplasm, and 27 cases of suspicious malignancy. Forty-seven cases (43.93%) were negative for CD56, including 17 cases of indeterminate lesion, 6 cases of follicular neoplasm, and 24 cases of suspicious malignancy. Sixty-four cases (59.81%) were positive for IHC-COMB1, including 23 cases of indeterminate lesion, 8 cases of follicular neoplasm, and 33 cases of suspicious malignancy. Seventy-six cases (71.03%) were positive for IHC-COMB2, including 32 cases of indeterminate lesion, 11 cases of follicular neoplasm, and 33 cases of suspicious malignancy. Ninety-five cases (88.79%) were positive for IHC-COMB3, including 38 cases of indeterminate lesion, 22 cases of follicular neoplasm, and 35 cases of suspicious malignancy (Table 4).

**Table 4:** The results of immunohistochemistry of CNB samples

IHC		Classification of CNB samples based on morphology, no			
Markers	Results	Indeterminate lesion	Follicular neoplasm	Suspicious for malignancy	Total
CK19	Negative	9	23	1	33
	Positive	31	9	34	74
Galectin-3	Negative	7	12	1	20
	Positive	33	20	34	87
HBME-1	Negative	16	25	8	49
	Positive	24	7	27	58
CD56	Positive	23	26	11	60
	Negative	17	6	24	47
IHC-COMB1	Negative	17	24	2	43
	Positive	23	8	33	64
IHC-COMB2	Negative	8	21	2	31
	Positive	32	11	33	76
IHC-COMB3	Negative	2	10	0	12
	Positive	38	22	35	95
Total		40	32	35	107

*CNB*, core needle biopsy; *IHC*, immunohistochemistry; *IHC-COMB1*, CD56 negative no matter whether CK19, galectin-3, and HBME-1 are positive or not/CD56 positive and all of CK19, galectin-3, and HBME-1 simultaneously positive; *IHC-COMB2*, CD56 negative no matter whether CK19, galectin-3, and HBME-1 are positive or not/CD56 positive and at least two of CK19, galectin-3, and HBME-1 simultaneously positive; *IHC-COMB3*, CD56 negative no matter whether CK19, galectin-3, and HBME-1 are positive or not/CD56 positive and at least one of CK19, galectin-3, and HBME-1 simultaneously positive

### Results of NGS

Sixty-eight cases (63.55%) were positive for NGS. The 41 cases with BRAF V600E mutation included 12 cases of indeterminate lesions and 29 cases of suspicious malignancy. The 8 cases with RAS mutation included 3 cases of indeterminate lesion and 5 cases of follicular neoplasm. The 7 cases with RET fusion included 5 cases of indeterminate lesion, 1 case of follicular neoplasm, and 1 case of suspicious malignancy. All of the 4 cases with NTRK fusion were indeterminate lesions. The one case with ALK fusion was indeterminate lesion. All of the 4 cases with TERT mutation were follicular neoplasm. The rest of the three follicular neoplasms had PTEN mutation, PPARγ fusion, and non-V600E BRAF mutation separately (Table 5).

**Table 5:** The results of OncoAim®-NGS of CNB samples

NGS	Classification of CNB samples based on morphology, no			
	Indeterminate lesion	Follicular neoplasm	Suspicious for malignancy	Total
Negative	15	19	5	39
BRAF V600E	12	0	29	41
PTEN mutation	0	1	0	1
ALK fusion	1	0	0	1
RET fusion	5	1	1	7
NTRK fusion	4	0	0	4
RAS mutation	3	5	0	8
PPARγ fusion	0	1	0	1
TERT mutation	0	4	0	4
Non-V600E BRAF mutation	0	1	0	1
Total	40	32	35	107

*CNB*, core needle biopsy; *NGS*, next-generation sequencing

### Results of integrated IHC-NGS

Ninety-nine cases (92.52%) were positive for the integrated IHC-NGS, including 38 cases of indeterminate lesion, 26 cases of follicular neoplasm, and 35 cases of suspicious malignancy.

### Comparison between biomarkers’ results of CNB samples and classification of matched resected specimens

**Fig. 1:**
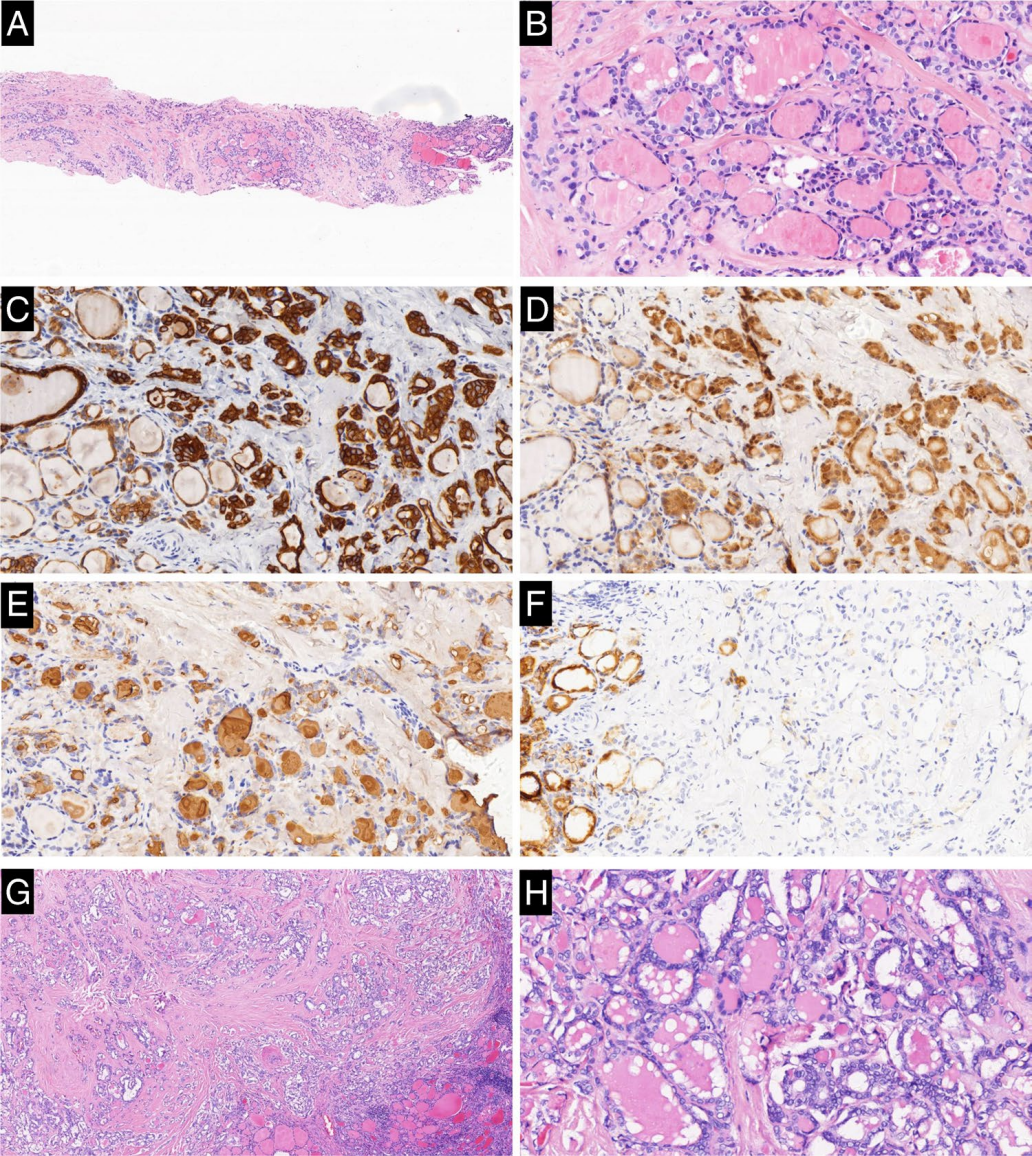
Case classified as indeterminate lesion in the CNB sample while follicular variant of papillary thyroid carcinoma in the matched resected specimen. **a** Tumor in the CNB sample is entirely composed of follicular structures (H&E × 40). **b** High magnification of the lesion shows the follicular structures lined by cells with nuclei scored 1 (H&E × 200). **c** Cytoplasm and membrane of tumor cells in the CNB sample are diffusely reactive for CK19 with strong intensity, while the normal follicular cells are reactive with weak intensity (CK19 × 200). **d** Cytoplasm and nuclei of tumor cells in the CNB sample are diffusely reactive for galectin-3 with strong intensity, while the normal follicular cells are nonreactive (galectin-3 × 200). **e** Membranes of tumor cells in the CNB sample are partially (about 30%) reactive for HBME-1 with intermediate intensity, while the normal follicular cells are nonreactive (HBME-1 × 200). **f** Tumor cells in the CNB samples are nonreactive for CD56, while membrane and cytoplasm of the normal follicular cells are diffusely reactive with strong intensity (CD56 × 200). **g** Tumor in the matched resected specimen is entirely composed of follicular structures. (H&E × 40). **h** High magnification of the lesion shows the follicular structures lined by cells with nuclei scored 3 (H&E × 200)

**Fig. 2:**
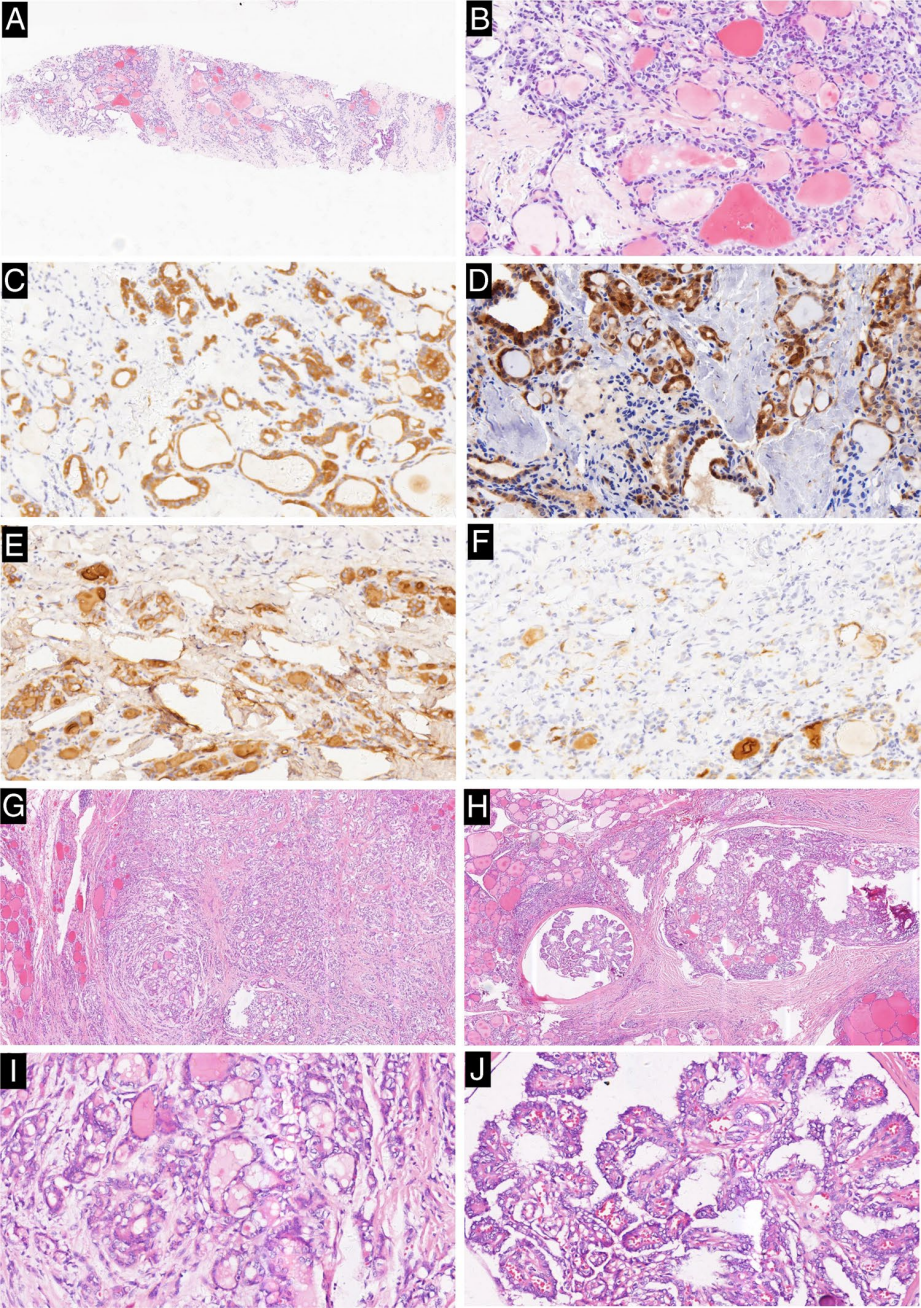
Case classified as indeterminate lesion in the CNB sample while conventional papillary thyroid carcinoma with a follicular predominant growth pattern in the matched resected specimen. **a** Tumor in the CNB sample is entirely composed of follicular structures (H&E × 40). **b** High magnification of the lesion shows the follicular structures lined by cells with nuclei scored 2 (H&E × 200). **c** Cytoplasm and membrane of tumor cells in the CNB sample are diffusely reactive for CK19 with strong intensity, while the normal follicular cells are nonreactive (CK19 × 200). **d** Cytoplasm and nuclei of tumor cells in the CNB sample are diffusely reactive for galectin-3 with strong intensity, while the normal follicular cells are nonreactive (galectin-3 × 200). **e** Membranes of tumor cells in the CNB sample are diffusely reactive for HBME-1 with strong intensity, while the normal follicular cells are nonreactive (HBME-1 × 200). **f** Tumor cells in the CNB samples are nonreactive for CD56, while membrane and cytoplasm of the normal follicular cells are diffusely reactive with intermediate intensity (CD56 × 200). **g**, **h**, Tumor in the matched resected specimen is almost entirely composed of follicular structures, except of focal papillary structure (H&E × 40). **i** High magnification of the lesion shows the follicular structures lined by cells with nuclei scored 3 (H&E × 200). **j** High magnification of the lesion shows the papillary structures lined by cells with nuclei scored 3 (H&E × 200)

**Fig. 3:**
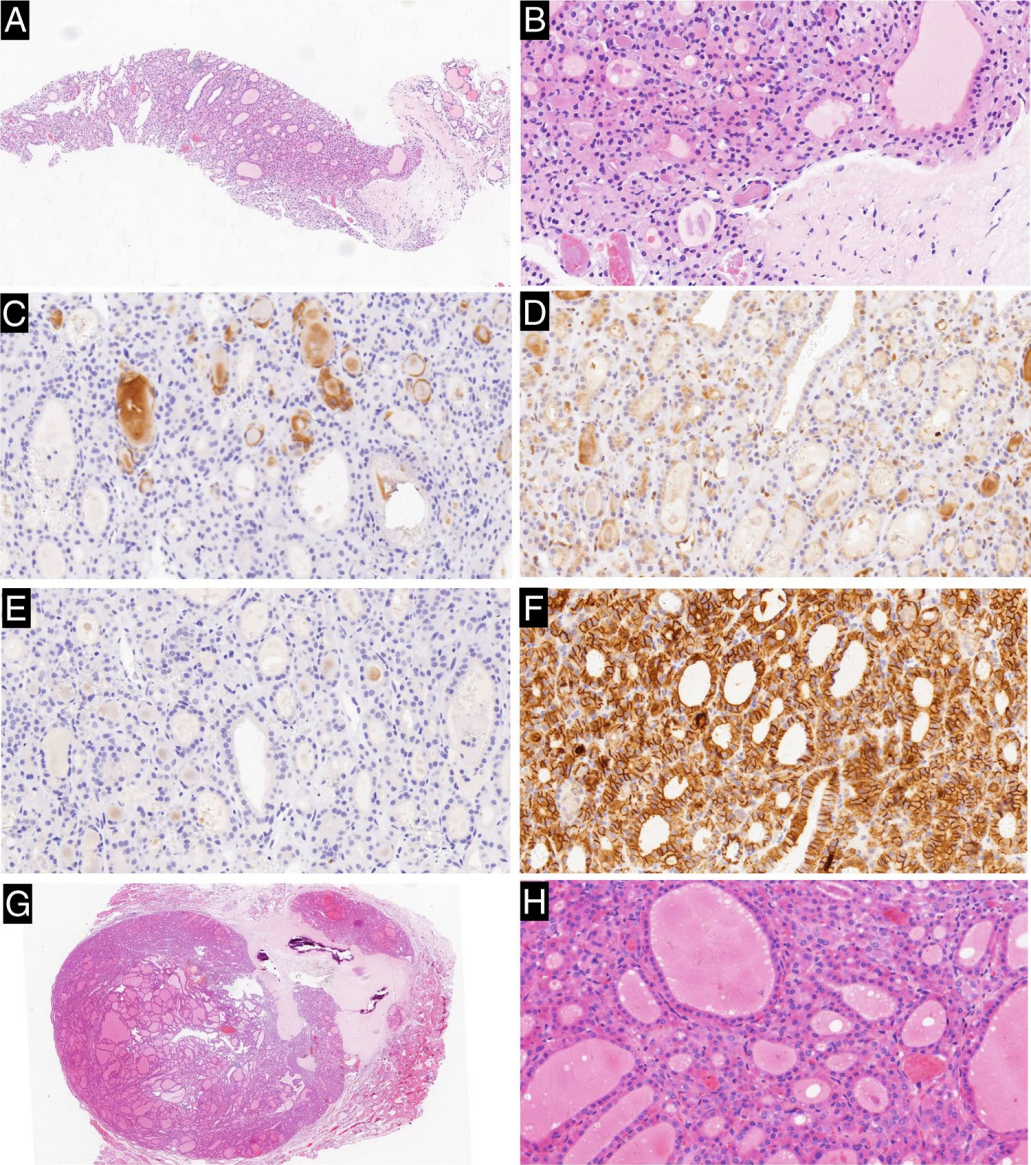
Case classified as follicular neoplasm in the CNB sample while follicular thyroid carcinoma in the matched resected specimen. **a** The CNB specimen shows a microfollicular proliferative lesion with a thick fibrous capsule separating it from the normal follicles (H&E × 40). **b** High magnification of the lesion shows the follicular structures lined by cells with nuclei scored 0 (H&E × 200). **c** Tumor cells in the CNB sample are negative for CK19 (CK19 × 200). **d** Tumor cells in the CNB sample are negative for galectin-3 (galectin-3 × 200). **e** Tumor cells in the CNB sample are negative for HBME-1 (HBME-1 × 200). **f** Tumor cells in the CNB samples are positive for CD56 (CD56 × 200). **g** Tumor in the matched resected specimen is encapsulated with capsular infiltration and entirely composed of follicular structures (H&E × 40). **h** High magnification of the lesion shows the follicular structures lined by cells with nuclei scored 0 (H&E × 200)

Of the 74 cases positive of CK19 on CNB samples, 66 were classified as malignant and 8 were classified as benign on the matched resected samples. Of the 87 cases positive of galectin-3 on CNB samples, 76 were classified as malignant and 11 were classified as benign on the matched resected samples. Of the 58 cases positive of HBME-1 on CNB samples, 54 were classified as malignant and 4 were classified as benign on the matched resected samples. All 47 cases of CD56 negative on CNB samples were diagnosed as malignant on the matched resected sample too (Table 6) (Figs. 1, 2, and 3).

**Table 6:** Predictive value of biomarkers for all cases

	CNB samples, no	Matched resected specimens, no			Predictive value, %				
		Benignity	Malignancy	Total	Sen	Spe	PPV	NPV	AC
CK19	Negative	14	19	33	77.65	63.63	89.19	42.42	74.77
	Positive	8	66	74					
Galectin-3	Negative	11	9	20	89.41	50.00	87.36	55.00	81.31
	Positive	11	76	87					
HBME-1	Negative	18	31	49	63.53	81.81	93.10	36.73	67.29
	Positive	4	54	58					
CD56	Positive	22	38	60	55.29	100	100	36.67	64.49
	Negative	0	47	47					
IHC-COMB1	Negative	20	23	43	72.94	90.91	96.88	46.51	76.64
	Positive	2	62	64					
IHC-COMB2	Negative	15	16	31	81.18	68.18	90.79	48.39	78.50
	Positive	7	69	76					
IHC-COMB3	Negative	8	4	12	95.29	36.36	85.26	66.67	83.18
	Positive	14	81	95					
NGS	Negative	20	17	37	80.00	90.90	97.14	54.05	82.24
	Positive	2	68	70					
IHC-NGS	Negative	8	0	8	100	36.36	85.86	100	86.92
	Positive	14	85	99					
	Total	22	85	107					

*CNB*, core needle biopsy; *Sen*, sensitivity; *Spe*, specificity; *PPV*, positive predictive value; *NPV*, negative predictive value; *AC*, accuracy; *IHC-COMB1*, CD56 negative no matter whether CK19, galectin-3, and HBME-1 are positive or not/CD56 positive and all of CK19, galectin-3, and HBME-1 simultaneously positive; *IHC-COMB2*, CD56 negative no matter whether CK19, galectin-3, and HBME-1 are positive or not/CD56 positive and at least two of CK19, galectin-3, and HBME-1 simultaneously positive; *IHC-COMB3*, CD56 negative no matter whether CK19, galectin-3, and HBME-1 are positive or not/CD56 positive and at least one of CK19, galectin-3, and HBME-1 simultaneously positive; *IHC-NGS*, immunohistochemistry and next-generation sequencing combination

Of the 64 cases of IHC-COMB1 positive on CNB samples, 62 were classified as malignant and two were classified as benign on the matched resected samples. Of the 76 cases of IHC-COMB2 positive on CNB samples, 69 were classified as malignant and seven were classified as benign on the matched resected samples. Of the 95 cases of IHC-COMB3 positive on CNB samples, 81 were classified as malignant and 14 were classified as benign on the matched resected specimens (Table 6).

Of the 70 cases of NGS positive on CNB samples, 68 were classified as malignant and two were classified as benign on the matched resected specimens (Table 6). The 41 cases with BRAF V600E mutation included 29 cases of CPTC and 12 cases of FVPTC. The 8 cases with RAS mutation included 5 cases of FTC, 2 cases of FVPTC, and 1 case of NH. The 7 cases with RET fusion included 5 cases of FVPTC, 1 case of CPTC, and 1 case of FTC. All of the 4 cases with NTRK fusion were FVPTC. All of the 4 cases with TERT mutation were FTC. The one case with ALK fusion was FVPTC. The one case with PTEN mutation was FTC. The one case with PPARγ fusion was FTC. The one case with non-V600E BRAF mutation was FA.

Of the 99 cases positive of integrated IHC-NGS on CNB samples, 85 were classified as malignant and 14 were classified as benign on the matched resected samples (Table 6), including 35 cases of CPTC, 27 cases of FVPTC, 23 cases of FTC, 8 cases of NH, 3 cases of FA, and 3 cases of thyroiditis.

### Predictive value of biomarkers

Taking the classification of the matched resected specimens as the gold standard, the sensitivity, specificity, PPV, NPV, and accuracy for preoperative malignancy evaluation for the whole cohort were 77.65%, 63.64%, 89.19%, 42.43%, and 74.77% for CK19; 89.42%, 50.00%, 87.36%, 55.00%, and 81.31% for galectin-3; 63.53%, 81.82%, 93.10%, 36.73%, and 67.29% for HBME-1; 55.29%, 100.00%, 100.00%, 36.67%, and 64.49% for CD56; 80.00%, 90.91%, 97.14%, 54.05%, and 82.24% for NGS; 72.94%, 90.91%, 96.88%, 46.51%, and 76.64% for IHC-COMB1; 81.18%, 68.18%, 90.79%, 48.39%, and 78.50% for IHC-COMB2; 95.29%, 36.36%, 85.26%, 66.67%, and 83.18% for IHC-COMB3; and 100.00%, 36.36%, 85.86%, 100.00%, and 86.92% for integrated IHC-NGS (Table 6).

The sensitivity, specificity, PPV, NPV, and accuracy for preoperative malignancy evaluation of non-FN-lesions were 95.16%, 53.85%, 90.77%, 70.00%, and 88.00% for CK19; 95.16%, 38.46%, 88.06%, 62.50%, and 85.33% for galectin-3; 77.42%, 76.92%, 94.12%, 41.67%, and 58.00% for HBME-1; 66.13%, 100.00%, 100.00%, 38.24%, and 72.00% for CD56; 90.32%, 92.31%, 98.25%, 66.67%, and 90.67% for NGS; 88.71%, 92.30%, 98.21%, 63.16%, and 89.33% for IHC-COMB1; 96.77%, 61.54%, 92.31%, 80.00%, and 90.67% for IHC-COMB2; 100.00%, 15.38%, 84.93%, 100.00%, and 85.33% for IHC-COMB3; and 100.00%, 36.36%, 85.86%, 100.00%, and 86.92% for integrated IHC-NGS (Table 7).

**Table 7:** Predictive value of biomarkers for cases of non-follicular-neoplasm-lesion

	CNB samples, no	Matched resected samples, no			Predictive value, %				
		Benignity	Malignancy	Total	Sen	Spe	PPV	NPV	AC
CK19	Negative	7	3	10	95.16	53.85	90.77	70.00	88.00
	Positive	6	59	65					
Galectin-3	Negative	5	3	8	95.16	38.46	88.06	62.50	85.33
	Positive	8	59	67					
HBME-1	Negative	10	14	24	77.42	76.92	94.12	41.67	58.00
	Positive	3	48	51					
CD56	Positive	13	21	34	66.13	100	100	38.24	72.00
	Negative	0	41	41					
IHC-COMB1	Negative	12	7	19	88.71	92.30	98.21	63.16	89.33
	Positive	1	55	56					
IHC-COMB2	Negative	8	2	10	96.77	61.54	92.31	80.00	90.67
	Positive	5	60	65					
IHC-COMB3	Negative	2	0	2	100	15.38	84.93	100	85.33
	Positive	11	62	73					
NGS	Negative	12	6	18	90.32	92.31	98.25	66.67	90.67
	Positive	1	56	57					
IHC-NGS	Negative	2	0	2	100	15.38	84.93	100	85.33
	Positive	11	62	73					
	Total	13	62	75					

*CNB*, core needle biopsy; *Sen*, sensitivity; *Spe*, specificity; *PPV*, positive predictive value; *NPV*, negative predictive value; *AC*, accuracy; *IHC-COMB1*, CD56 negative no matter whether CK19, galectin-3, and HBME-1 are positive or not/CD56 positive and all of CK19, galectin-3, and HBME-1 simultaneously positive; *IHC-COMB2*, CD56 negative no matter whether CK19, galectin-3, and HBME-1 are positive or not/CD56 positive and at least two of CK19, galectin-3, and HBME-1 simultaneously positive; *IHC-COMB3*, CD56 negative no matter whether CK19, galectin-3, and HBME-1 are positive or not/CD56 positive and at least one of CK19, galectin-3, and HBME-1 simultaneously positive; *IHC-NGS*, immunohistochemistry and next-generation sequencing combination

The sensitivity, specificity, PPV, NPV, and accuracy for preoperative malignancy evaluation of FN were 30.43%, 77.77%, 77.77%, 30.43%, and 43.75% for CK19; 73.91%, 66.67%, 85.00%, 50.00%, and 71.88% for galectin-3; 26.09%, 88.89%, 85.71%, 32.00%, and 43.75% for HBME-1; 26.09%, 100.00%, 100.00%, 34.62%, and 46.88% for CD56; 52.17%, 88.89%, 92.31%, 42.11%, and 62.50% for NGS; 30.43%, 88.89%, 87.50%, 33.33%, and 46.88% for IHC-COMB1; 39.13%, 77.78%, 81.82%, 33.33%, and 50.00% for IHC-COMB2; 82.61%, 66.67%, 86.36%, 60.00%, and 78.13% for IHC-COMB3; and 100.00%, 66.67%, 88.46%, 100.00%, and 90.63% for integrated IHC-NGS integrated(Table 8).

**Table 8:** Predictive value of biomarkers for cases of follicular neoplasm

	CNB samples, no		Matched resected samples, no			Predictive value, %					
			Benignity	Malignancy	Total	Sen		Spe	PPV	NPV	AC
CK19		Negative	7	16	23		30.43	77.77	77.77	30.43	43.75
		Positive	2	7	9						
Galectin-3		Negative	6	6	12		73.91	66.67	85.00	50.00	71.88
		Positive	3	17	20						
HBME-1		Negative	8	17	25		26.09	88.89	85.71	32.00	43.75
		Positive	1	6	7						
CD56		Positive	9	17	26		26.09	100	100	34.62	46.88
		Negative	0	6	6						
IHC-COMB1		Negative	8	16	24		30.43	88.89	87.50	33.33	46.88
		Positive	1	7	8						
IHC-COMB2		Negative	7	14	21		39.13	77.78	81.82	33.33	50.00
		Positive	2	9	11						
IHC-COMB3		Negative	6	4	10		82.61	66.67	86.36	60.00	78.13
		Positive	3	19	22						
NGS		Negative	8	11	19		52.17	88.89	92.31	42.11	62.50
		Positive	1	12	13						
IHC-NGS		Negative	6	0	6		100	66.67	88.46	100	90.63
		Positive	3	23	26						
		Total	9	23	32						

*CNB*, core needle biopsy; *Sen*, sensitivity; *Spe*, specificity; *PPV*, positive predictive value; *NPV*, negative predictive value; *AC*, accuracy; *IHC-COMB1*, CD56 negative no matter whether CK19, galectin-3, and HBME-1 are positive or not/CD56 positive and all of CK19, galectin-3, and HBME-1 simultaneously positive; *IHC-COMB2*, CD56 negative no matter whether CK19, galectin-3, and HBME-1 are positive or not/CD56 positive and at least two of CK19, galectin-3, and HBME-1 simultaneously positive; *IHC-COMB3*, CD56 negative no matter whether CK19, galectin-3, and HBME-1 are positive or not/CD56 positive and at least one of CK19, galectin-3, and HBME-1 simultaneously positive; *IHC-NGS*, immunohistochemistry and next-generation sequencing combination

## Discussion

Morphological changes, including nuclear score, architecture (papillary or follicular), and growth pattern (infiltrative or encapsulated), are critical for diagnosing thyroid tumors. Based on the criteria above, major cases can be diagnosed undoubtedly. However, some cases are difficult to determine based on histological morphology alone. Compared to resected specimens, the diagnoses of biopsies are more challenging. The uncertain diagnosis rate is 10–40% for FNB and 5–20% for CNB [5]. Our comparative study between CNB and resected specimens of thyroid nodules showed that 74 of 578 cases could not be ascertained as malignant or benign based on the CNB sample’s morphology alone [6]. The reason is that only follicles visible on CNB with atypical nuclei without normal tissue as a background make it impossible to differentiate FTC, FVPTC, and CPTC with a follicular predominant growth pattern from FA, NH, and thyroiditis. Therefore, studying the application of biomarkers in distinguishing uncertain biopsy samples is necessary.

Immunohistochemistry is the most popular ancillary technique used in pathological practice. Studies on resected specimens showed that CK19, galectin-3, HBME-1, and CD56 were very helpful in discriminating malignancy from benignity [10–13]. In Dunderovic et al.’s study, the sensitivity of CK19, galectin-3, HBME-1, and CD56 was 75.41%, 88.52%, 71.31%, and 58.20%, respectively, and the specificity of CK19, galectin-3, HBME-1, and CD56 was 70.89%, 64.56%, 84.81%, and 92.41%, separately [10]. Based on the knowledge above, it was supposed that IHC might play a role in improving the accuracy of diagnosing uncertain biopsy samples. We searched papers published in English in PubMed and found only one focusing on this topic. In this paper, Song et al. reported that the continued uncertain rate was 42.9% for FNB and 11.3% for CNB after IHC was applied [14].

Our study showed that, taking the resected specimens’ diagnosis as the gold standard, biomarker’s efficiency in determining the uncertain CNB samples as malignant or benign was various. Besides, even the same marker had a different power between FN and non-FN-lesions. The specificity of CD56 is perfect (100%) for both FN and non-FN-lesions, but the sensitivity is low (66.13% for non-FN-lesions and 26.09% for FN). Therefore, CD56 negative is particular for “ruling in” the malignant CNB samples; however, CD56 positive should not be used as the indicator of benignity. On the contrary, galectin-3 showed high sensitivity (95.16%) for non-FN-lesions and moderate sensitivity (73.91%) for FN but low specificity (38.46% for non-FN-lesions and 66.67% for FN). Hence, galectin-3 negative could be highly suggestive of benignity for non-FN-lesions and cautiously used to support benignity for FN. Galectin-3 positive should not be used as the indicator of malignancy.

Given the limitation of a single marker, it is judicious to diagnose based on the integrated results. Considering that CD56 has perfect specificity but low sensitivity, the combination should precisely pick back the cases left out by CD56. Our study showed that keeping the CD56-negative cases in the cohort of malignancy and picking back the cases with CD56 positive and all of the other three markers simultaneously positive was a suitable strategy to balance the specificity (92.30%) and sensitivity (88.71%) for the non-FN-lesion. But for FN, none of the combined panels had apparent advantages over a single marker.

In the past 10 years, we have witnessed significant progress in the molecular pathology of thyroid carcinoma. In 2014, The Cancer Genome Atlas (TCGA) reported the comprehensive genomic characteristics of PTC. Ninety-seven percent of PTCs have unique molecular alterations, in which BRAF V600E mutations, RAS mutations, RET fusions, and TERT mutations are frequently detected, but EIF1AX mutations, ALK fusions, and NTRK1 or NTRK3 fusions are infrequent [15]. Subsequently, the genotypes of FTC, poorly differentiated thyroid carcinoma (PDTC), and anaplastic thyroid carcinoma (ATC) have also been reported. In FTC, RAS mutations, PPARγ fusions, and TERT mutations are frequently detected, but BRAF K601E mutations and EIF1AX mutations are infrequent. In PDTC and ATC, BRAF V600E mutations, RAS mutations, TERT mutations, and TP53 mutations are frequently detected [16–18]. Based on their understanding of thyroid carcinoma’s mutational profile, researchers have tried to use diverse molecular approaches to improve diagnosing uncertain biopsy samples and have presented various published results. The sensitivity and specificity of gene testing for discriminating malignancy from benignity were 63–94% and 52–99%, respectively, with FNB [19–21]. Regardless of how sensitive or specific it is, applying gene testing to FNB is inconvenient in clinical practice because specialized sample collection is required at the initial procedure. Besides, the morphology of FNB samples used in the molecular test is unknown. In contrast, CNB samples are routinely stored as paraffin-embedded blocks in which DNA can readily be extracted and morphology can be reviewed at any moment. In this case, gene testing is supposed to distinguish uncertain samples more practically and effectively on CNB than FNB.

Compared to FNB, the number of publications about CNB is minimal, and only a few single mutations have been reported [22–25]. In our research, uncertain CNB samples were detected by NGS using the commercial panel OncoAim®, which detected 26 genes covering the major molecular alterations of thyroid carcinoma. The sample was recorded as NGS positive when confirmed pathogenic or likely pathogenic mutations were detected. Taking the diagnosis of the resected specimens as the gold standard, NGS is highly specific (92.31%) and sensitive (90.32%) for the non-FN-lesion, and highly specific (88.89%) but low sensitive (52.17%) for the FN. In other words, NGS’s positive result suggests malignancy strongly for both non-FN and FN. But the negative result should be cautiously used as an indicator of benignity for non-FN-lesion and not be used as an indicator of benignity for FN. Taking PPV and NPV considered together, NGS’s efficiency was high for non-FN-lesion and moderate for FN.

Because NGS is not a universal technique and different laboratories may use diverse gene panels, platforms, and methods, the working power of NGS depends largely on each laboratory’s technical details. In practice, it is a suitable way for pathologists to interpret NGS results based on the knowledge integrating the literature’s reports and own lab’s data. All of the data and analysis about NGS in our research are based on the specific commercial tool OncoAim®.

In our study, there were 37 cases with NGS negative results on CNB samples. The diagnosis of their matched resected specimens was benign for 20 cases and malignant for 17 cases. The 17 malignant cases included 11 FTC cases, 5 CPTC cases, and 1 FVPTC case. Then, we detected the 17 cases’ genes on matched resected specimens and found that 11 FTC cases were really negative and the other six were false negative, including 5 CPTC cases with BRAF V600E and one FVPTC with NRAS mutation. Furthermore, the CNB slides of the six false-negative cases were reviewed, and it is shown that very few tumor components (less than 5%) are in them. In conclusion, the inherent features of gene mutations of thyroid tumors, especially follicular neoplasm, are considered the main reason for NGS’s relatively low efficiency as a benign marker. The false-negative results due to the limitation of tumor quantity in CNB samples are another factor in weakening NGS’s power to pick up benign cases, even though the influence is lower than FNB.

Fortunately, all six NGS false-negative cases were positive for CK19, galectin-3, and HBME-1 and negative for CD56 on CNB, which gave us the confidence to make a malignant diagnosis. So, IHC plays an essential role in these cases with NGS’s false-negative results due to the limitation of tumor quantity in CNB samples.

For non-FN-lesions, either IHC or NGS can work well individually. Therefore, combining them is unnecessary and not cost-effective. On the contrary, neither of them is powerful enough for FN when used separately. So, designing an integrated panel for improving the predictive value is a practical need. Considering the treatment of FN recommended by the NCCN guideline [26], patients may benefit more from the safe “rule out” strategy than the precise “rule in” strategy. Based on this principle, we designed the integrated panel to keep the NGS positive cases in the cohort of malignancy and pick back the cases with at least one of four IHC markers positive. This panel can raise sensitivity and NPV to 100% and keep acceptable specificity (66.67%) and PPV (88.46%), which may be superior to use IHC or NGS separately. The negative FN cases are highly possible to be benign, and nodule surveillance may be recommended with a bit of worry.

Finally, although it is acknowledged that presenting the results as a risk of malignancy (ROM) than a binary fashion is more clinically valuable, such modification of ROM is currently unavailable due to limited number of cases. Hence, further research is required to explore the application of biomarkers in evaluating the ROM of uncertain samples.

## Conclusions

The application of biomarkers in distinguishing uncertain CNB samples of thyroid nodules is available and capable. CD56 negative or NGS positive suggests malignancy strongly for both FN and non-FN-lesions, which may be used as a “rule in” tool. The negativity of the integrated IHC and the integrated IHC-NGS implies a high possibility to be benign for non-FN-lesions and FN separately, which can work as a “rule out” tool. Considering the balance of specificity and sensitivity, NGS is the best for non-FN-lesions and the integrated IHC-NGS is the best for FN.

Because NGS is not a universal technique, the working power of NGS depends largely on each laboratory’s technical details. Pathologists should interpret NGS results based on the knowledge integrating the literature’s reports and own lab’s data. All of the data and analysis about NGS in our research are based on the specific commercial tool OncoAim®.

## Data Availability

The datasets used and analyzed during the current study are available from the corresponding author on reasonable request.

## Abbreviations

CNB: Core needle biopsy
FNB: Fine-needle biopsy
H&E: Hematoxylin and eosin
IHC: Immunohistochemistry
NGS: Next-generation sequencing
IHC-COMB: Immunohistochemistry combination
IHC-NGS: Immunohistochemistry and next-generation sequencing combination
PPV: Positive predictive value
NPV: Negative predictive value
NH: Nodular hyperplasia
FA: Follicular adenoma
FTC: Follicular thyroid carcinoma
PTC: Papillary thyroid carcinoma
FVPTC: Follicular variant of papillary thyroid carcinoma
CPTC: Conventional papillary thyroid carcinoma
FN: Follicular neoplasm
TCGA: The Cancer Genome Atlas
PDTC: Poorly differentiated thyroid carcinoma
ATC: Anaplastic thyroid carcinoma
